# The SWITCH algorithm: An expert consensus on treat‐to‐target criteria for chronic prurigo

**DOI:** 10.1111/jdv.70171

**Published:** 2025-11-08

**Authors:** Lea S. Stahl, Claudia Zeidler, Joyce Loewenthal, Svenja Royeck, Matthias Augustin, Robert Bissonnette, Sarina Elmariah, Brian S. Kim, Laurent Misery, Hiroyuki Murota, Hong Liang Tey, Elke Weisshaar, Gil Yosipovitch, Franz J. Legat, Adam Reich, Martin Metz, Sonja Ständer

**Affiliations:** ^1^ Section Pruritus Medicine, Department of Dermatology, and Center of Chronic Pruritus University Hospital Münster Münster Germany; ^2^ Competence Center of Healthcare Research in Dermatology (CVderm), Institute for Health Services Research in Dermatology and Nursing (IVDP) University Medical Center Hamburg‐Eppendorf Hamburg Germany; ^3^ Innovaderm Research Montreal Quebec Canada; ^4^ Department of Dermatology University of California San Francisco California USA; ^5^ Department of Dermatology Icahn School of Medicine at Mount Sinai New York New York USA; ^6^ Laboratoire Interactions Neurones‐Keratinocytes (LINK) University of Brest Brest France; ^7^ Department of Dermatology and Venereology University Hospital of Brest Brest France; ^8^ Department of Dermatology Nagasaki University Graduate School of Biomedical Sciences Nagasaki Japan; ^9^ Department of Dermatology National Skin Center Singapore Singapore; ^10^ Division of Occupational Dermatology, Department of Dermatology University of Heidelberg Heidelberg Germany; ^11^ Miami Itch Center, Phillip Frost Department of Dermatology and Cutaneous Surgery, Miller School of Medicine University of Miami Miami Florida USA; ^12^ Department of Dermatology and Venereology Medical University of Graz Graz Austria; ^13^ Department of Dermatology University of Rzeszów Rzeszów Poland; ^14^ Institute of Allergology, Charité ‐ Universitätsmedizin Berlin Corporate Member of Freie Universität Berlin and Humboldt‐Universität Zu Berlin Berlin Germany; ^15^ Fraunhofer Institute for Translational Medicine and Pharmacology ITMP, Immunology and Allergology Berlin Germany

**Keywords:** chronic prurigo, chronic pruritus, itch, prurigo nodularis, treatment algorithm, treat‐to‐target

## Abstract

**Background:**

To date, the management of chronic prurigo (CPG; prurigo nodularis) relies on expert consensus and guidelines without providing detailed recommendations on long‐term treatment management.

**Objectives:**

This study was initiated by an expert committee to define, validate and reach consensus on treat‐to‐target (T2T) criteria and a decision‐supporting treatment algorithm for CPG.

**Methods:**

A prospective, single‐centre, non‐interventional study was conducted in adult CPG patients at a moderate‐to‐severe disease stage, experiencing intense itch (NRS ≥7; Group A), and in patients who considered themselves successfully treated (Group B). The patients answered questions about their most severe symptoms and essential therapy goals. A committee of 14 field experts evaluated the study results, identified outcome tools from clinical trials and defined and weighted T2T criteria. Based on this, a treatment algorithm was developed through an iterative consensus‐building method and anonymous voting.

**Results:**

171 patients (Group A, *n* = 96; Group B, *n* = 75) were interviewed. Itch (99.4%), the need to scratch (48%), and pruriginous lesions (45.6%) were the most frequently reported severe symptoms. Pruritus relief (over complete pruritus resolution) and healing of lesions were the key therapeutic goals. In clinical trials, the most frequently used instruments were assessments of worst/peak itch intensity, disease severity via IGA, DLQI and sleep quality. According to the results, the experts defined initial itch control as NRS ≤3, lesion healing with IGA‐S 0/1, and burden, along with their respective clinically measurable parameters, as T2T criteria. These criteria, with predefined cut‐off values, serve as the decision‐tree keys that constitute the treatment algorithm presented here, facilitating a decision to maintain or switch the treatment.

**Conclusions:**

The T2T criteria and the SWITCH treatment algorithm offer a valid and systematic method for evaluating and refining CPG treatment based on patient‐reported and expert‐validated data. This will help to improve the treatment of patients with CPG.

## Plain Language Summary

Chronic prurigo (also known as prurigo nodularis) is a bothersome skin condition characterized by persistent, itchy nodules that are difficult to treat. New therapies are available, but there are no established criteria for changing therapy.

To define and align on criteria and an approach for making decisions in the treatment of chronic prurigo, international experts conducted this study. Over 170 patients were interviewed about their treatment goals, including the criteria and expected timeframe for a successful therapy.

Two groups of patients were included in the study: one group that suffered from severe chronic prurigo and another that had been successfully treated. For the patients, itch relief and healing of the skin lesions were the key therapeutic goals. Using measurable parameters to quantify these goals, the expert committee defined criteria that must be achieved in the treatment and criteria for switching the therapy. Accordingly, and applying a stepwise agreement‐building method and anonymous voting, the experts developed a treatment algorithm, which they called the SWITCH algorithm. This algorithm will help improve the medical management of chronic prurigo.

## Graphical abstract

The SWITCH project assessed patients' most distressing symptoms and therapy goals, followed by an expert‐based consensus procedure to define T2T criteria for patients with chronic prurigo (CPG)/prurigo nodularis. Based on these validated criteria, a 4‐step SWITCH algorithm was established to enhance CPG therapy outcomes and reduce patient burden.

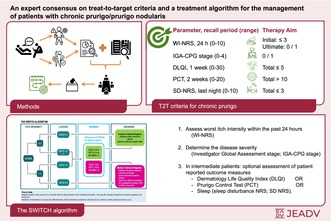

**Why was the study undertaken?**

- No treat‐to‐target criteria or algorithms for treating chronic prurigo/prurigo nodularis are available.

**What does this study add?**

- Clinically measurable treat‐to‐target criteria are defined, and based on these, a chronic prurigo treatment algorithm is presented.

**What are the implications of this study for disease understanding and/or clinical care?**

- The algorithm offers a valid and systematic approach to evaluating and adjusting treatment based on patient‐reported and expert‐validated data.

## INTRODUCTION

Chronic prurigo (CPG) is a burdensome dermatological condition characterized by chronic pruritus (≥6 weeks), persistent pruriginous nodules, papules and/or plaques, recurrent itch‐scratch cycles, sleep disturbances and a considerable impact on quality of life.^1^, ^2^ Prurigo nodularis (PN, also known as chronic nodular prurigo) is the predominant subtype of CPG.^1^, ^3^ CPG is usually resistant to traditional therapies, leading to a long‐lasting high patient burden.^4^ Recently, two biologics (Dupilumab, Nemolizumab) have been approved for this indication, which reduce pruritus intensity and skin lesions, resulting in a significant improvement in quality of life.^5^, ^6^ Accordingly, the medical care for this disease has substantially improved in the last 3 years.

However, besides an effective therapy, decision criteria for initiation, discontinuation and switch of the treatment as well as consideration of physician‐ and patient‐defined treatment goals are essential. These are all lacking in the currently available consensus paper and guidelines,^4^, ^7^ as neither treatment response nor treat‐to‐target (T2T) criteria have been defined yet. A T2T approach determines clear therapeutic objectives within a specified timeline after treatment initiation.^8^, ^9^ This strategy ensures that efficacy is assessed within a predetermined timeframe to guide further clinical decisions. In the management of CPG, a significant challenge lies in clearly defining treatment goals and outlining suitable strategies to achieve them. Consequently, treatment decisions are being made so far on a case‐by‐case basis, based on their efficacy or side effects, or due to economic reasons, the individual patient's situation (age, comorbidities) or their preferences.^10^ As a result, patients are often ineffectively treated, suffer for years and are not satisfied.^11^

In this study, 14 global experts gathered to agree on T2T criteria for adult patients with mild, moderate and severe CPG, including PN. Based on these criteria, a treatment algorithm for continuing or switching CPG therapy was developed.

## MATERIALS AND METHODS

An expert‐based consensus procedure, conducted within a nominal group process, was used to establish T2T criteria and a therapeutic algorithm for CPG, including PN, based on study outcomes and real‐world data. The process unfolded in parallel across several steps, including the formation of an international expert committee (EC), a comprehensive review of trials and their endpoints, evaluation of patients' treatment goals and management expectations and an expert discussion, definition, selection and voting‐based consensus for T2T criteria. A search in the Prospero database did not unveil any similar projects under the terms ‘prurigo AND therapy’, ‘prurigo AND DELPHI’, ‘prurigo AND algorithm’, ‘prurigo AND dupilumab’ and ‘prurigo AND criteria’. An IRB approval was obtained from the local ethics committee (2023‐389‐f‐S).

### Expert committee formation

The EC consisted of 14 leading dermatologists and CPG experts from Europe (M.A., F.L., L.M., M.M., A.R., S.St., E.W., C.Z.), the USA (S.E., B.K., G.Y.), Canada (R.B.) and Asia (H.M., H.L.T.). The EC members were invited based on their expertise in CPG treatment and related clinical trials, and their involvement in prior consensus efforts in dermatology. The EC was responsible for deciding on selected T2T criteria through digital discussions and electronic anonymous voting via www.Umfrageonline.com (enuvo GmbH). The coordinating team (L.S.S., J.L., S.R.) prepared the T2T criteria, conducted a comprehensive review of trial endpoints, prepared electronic surveys and interviewed patients.

### Comprehensive review

To identify the most used outcome tools in CPG Randomized Controlled Trials (RCTs), a comprehensive search was conducted in clinicaltrials.gov. The search was performed on 19 September 2023, to ensure the inclusion of the latest studies at that time. The terms ‘chronic prurigo’, ‘prurigo nodularis’ and ‘prurigo’ were assigned to the fields ‘condition/disease’ and ‘other’. The filters applied were RCTs and CPG/PN without any time limits. For each identified study, the primary and secondary endpoints were categorized according to the type of outcome (e.g. clinical outcomes, patient‐reported outcomes) and tabulated to determine those most used.

### Structured patient interview

The expectations of CPG patients were assessed using a structured interview. Patients (minimum 150 or until saturation of responses) were recruited between November 2023 and July 2024 in the Center of Chronic Pruritus, Department of Dermatology, University Hospital Münster, Germany and stratified into two groups based on their self‐assessed treatment success: Group A (Therapy Need) with moderate‐to‐severe CPG experiencing intense itch (worst itch numerical rating scale [WI‐NRS] ≥ 7) and Group B (Therapy Success) considering themselves successfully treated without predetermination of Chronic Prurigo Investigator Global Assessment for the stage (IGA‐CPG‐S) or NRS (to reflect real‐world perception). After giving informed consent the patients received a printed form of 12 questions addressing current treatment, current WI‐NRS, average itch NRS, worst itch VAS, average itch VAS, sleep disturbance, most severe symptoms of CPG (open question; ranking of predefined symptoms), highest therapy aim (open and closed questions; including time needed; ranking of aims), itch relief expectations (4 points; NRS 0/1) and lesion healing expectation. Further patient‐reported outcomes covered Prurigo Control Test (PCT), Dermatology Life Quality Index (DLQI) and ItchyQol. The physician documented demographics, history and current IGA for CPG.

At the time of the study, no scale domain interpretation for the sleep disturbance NRS was available. Hence, 51 independent CPG patients were recruited at the Center of Chronic Pruritus, Department of Dermatology, University Hospital Münster, Germany between February and April 2025. All these patients were interviewed for sleep disturbances using the corresponding NRS (NRS‐SD) to determine whether the disturbances were subjectively significant and rated the severity on a verbal rating scale (VRS) ranging from 0 to 4.

### Data collection and statistical analysis

Results of patient interviews were transferred weekly into an SPSS data sheet. The data were stored locally on the server of the University Hospital Münster. Descriptive statistics were used to summarize demographic and background characteristics of the sample, including means, standard deviations and proportions. Group comparisons were conducted using independent samples *t*‐tests for continuous variables and chi‐squared tests for categorical variables. Statistical significance was set at *p* < 0.05. All analyses were performed using SPSS Version 29 (IBM Corp., Armonk, NY).

## RESULTS

### Expert committee formation and review of RCT outcome tools

Between September 2023 and January 2025, the EC held five digital meetings. In the first meeting, the results of the RCT review were presented (Table S1). In Phase II and III studies, the most commonly assessed outcomes were pruritus intensity and disease severity. Itch served largely as the primary endpoint and was assessed mainly as worst itch or peak pruritus on the NRS (WI‐NRS). The IGA usually assessed disease severity (stage) for CPG, with IGA‐stages 3 and 4 reflecting moderate and severe CPG as inclusion criteria. Less frequently used outcomes included the Prurigo Activity and Severity (PAS) Score, Skin Pain NRS, DLQI and SD‐NRS. A few RCTs incorporated EQ‐5D for measuring health‐related quality of life, PCT and assessed the sleep onset latency and wakefulness after sleep onset.

Based on these results and on its own experience, the EC named and discussed initial T2T criteria, which were programmed into the electronic platform. The EC voted anonymously between October and November 2023 and considered the WI‐NRS/24 h (85.7%), the WI‐VAS/24 h (64.2%), the IGA‐CPG‐Stage (85.7%), the IGA‐CPG‐Activity (71.4%) and the PAS Score (64.3%) as the best instruments for making treatment decisions in CPG (Table S2).

### Structured patient interview

At the second meeting, the EC agreed to evaluate the relationship between selected instruments for treatment outcomes and patient expectations and finalized the patient questionnaire. Between November 2023 and July 2024, 171 patients were recruited and interviewed (96 Group A and 75 Group B). All baseline characteristics are shown in Table S3. The cohort was predominantly Caucasian (98.8%), of skin type II (87.1%). There was no significant difference in gender distribution and age between the two groups. The median age was 65 years (57.73). Groups A and B differed in itch intensity, quality of life impairment, prurigo control and disease severity. For example, Group A reported a significantly higher itch intensity (WI‐NRS/24 h = 8.2 ± 1.1) than Group B (2.7 ± 1.6; *p* < 0.001).

**FIGURE 1:**
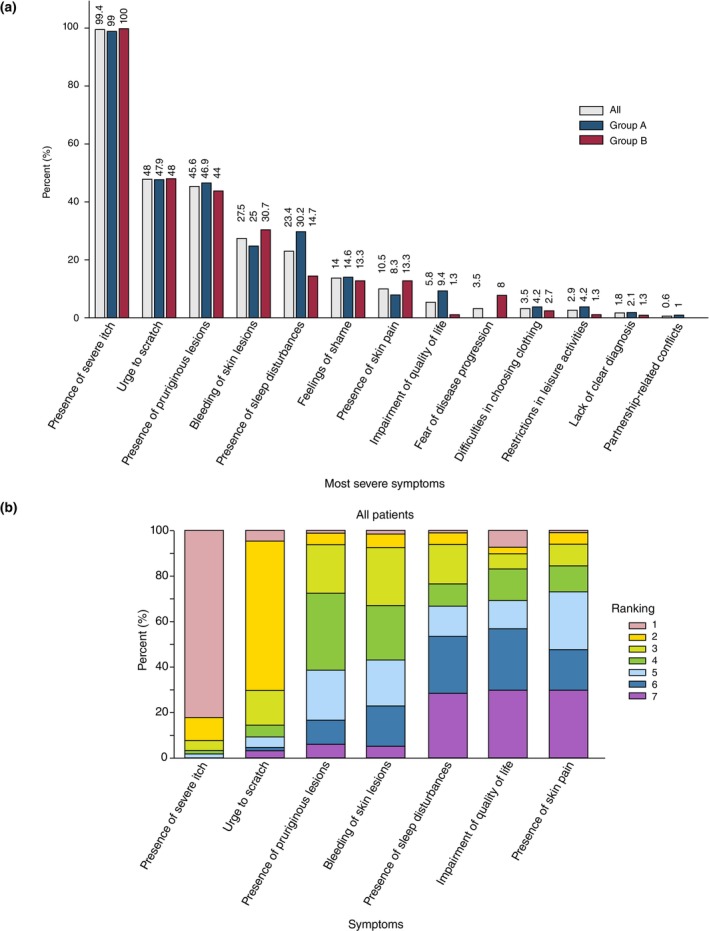
Most severe symptoms of chronic prurigo as reported by all patients (*n* = 171) including results from Group A (*n* = 96; Therapy Need) and Group B (*n* = 75; Therapy Success). (a) Open‐ended questions with self‐reporting of patients. (b) Ranking of seven predefined, most distressing symptoms (all patients). Patients were asked to rank in order of most distressing symptom from 1 (most important) to 7 and to assign each number 1–7 only once.

**FIGURE 2:**
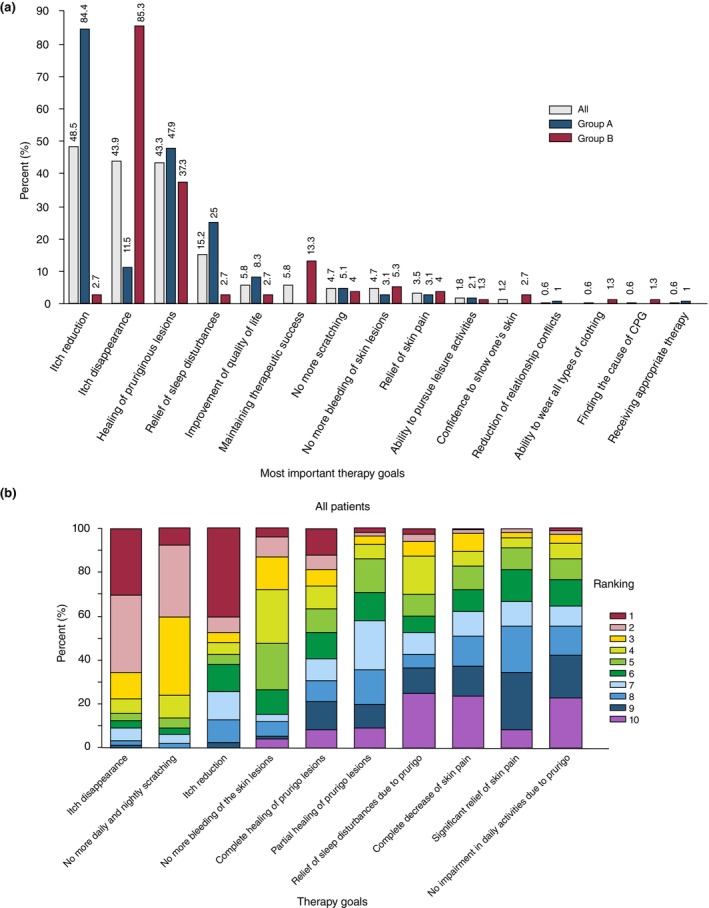
Most important therapy goals of chronic prurigo as reported by all patients (*n* = 171), including results from Group A (*n* = 96; Therapy Need) and Group B (*n* = 75; Therapy Success), based on open‐ended questions (a). (b) Ranking of 10 predefined treatment goals (all patients). Patients were asked to rank in order of most important goal from 1 (most important) to 10 (least important) and to assign each number 1–10 only once.

In open interviews as well as when selecting from a list of seven predefined symptoms assembled according to a previous publication,^12^ all patients taken together ranked itch (99.4% in open interviews/82.5% in ranking of symptoms), followed by the need to scratch (48% open interviews/66.1% ranking) and presence (45.6%/21.1%) and bleeding (27.5%/25.1%) of pruriginous lesions as their most distressing symptoms of the disease (Figure 1). On an open‐ended question, the most frequently mentioned therapy goals by all patients were pruritus reduction (48.5%) or complete itch disappearance (43.9%), followed by healing of pruriginous lesions (43.3%; Figure 2a). Among the 10 predefined treatment aims, 70.8% of all patients ranked pruritus treatment as their top priority, followed by the interruption of scratching (32.7%; Figure 2b).

In the additional group of CPG patients interviewed for sleep disturbances [*n* = 102; male, *n* = 50 (49.02%); female, *n* = 52 (50.98%); age, 58.14 (±14.74)], 50 participants reported subjectively significant sleep disturbances. In this group, the mean SD‐NRS score was 4.5 (±2.9). In contrast, individuals who did not report meaningful sleep disturbances (*n* = 52) had a lower mean SD‐NRS score of 1.3 (±2.0). Depending on patients' self‐assessed severity of sleep disturbance using a VRS, those reporting ‘very severe sleep loss’ (*n* = 2) had a mean SD‐NRS score of 9.0 (±0), with ‘severe sleep loss’ (*n* = 10) 6.4 (±2.7), with ‘moderate sleep loss’ (*n* = 28) 4.2 (±1.8), with ‘minimal sleep loss’ (*n* = 15) 1.7 (±1.2) and those with ‘no sleep loss’ (*n* = 38) 0.4 (±1.3).

### Definition of treat‐to‐target criteria and treatment algorithm

**FIGURE 3:**
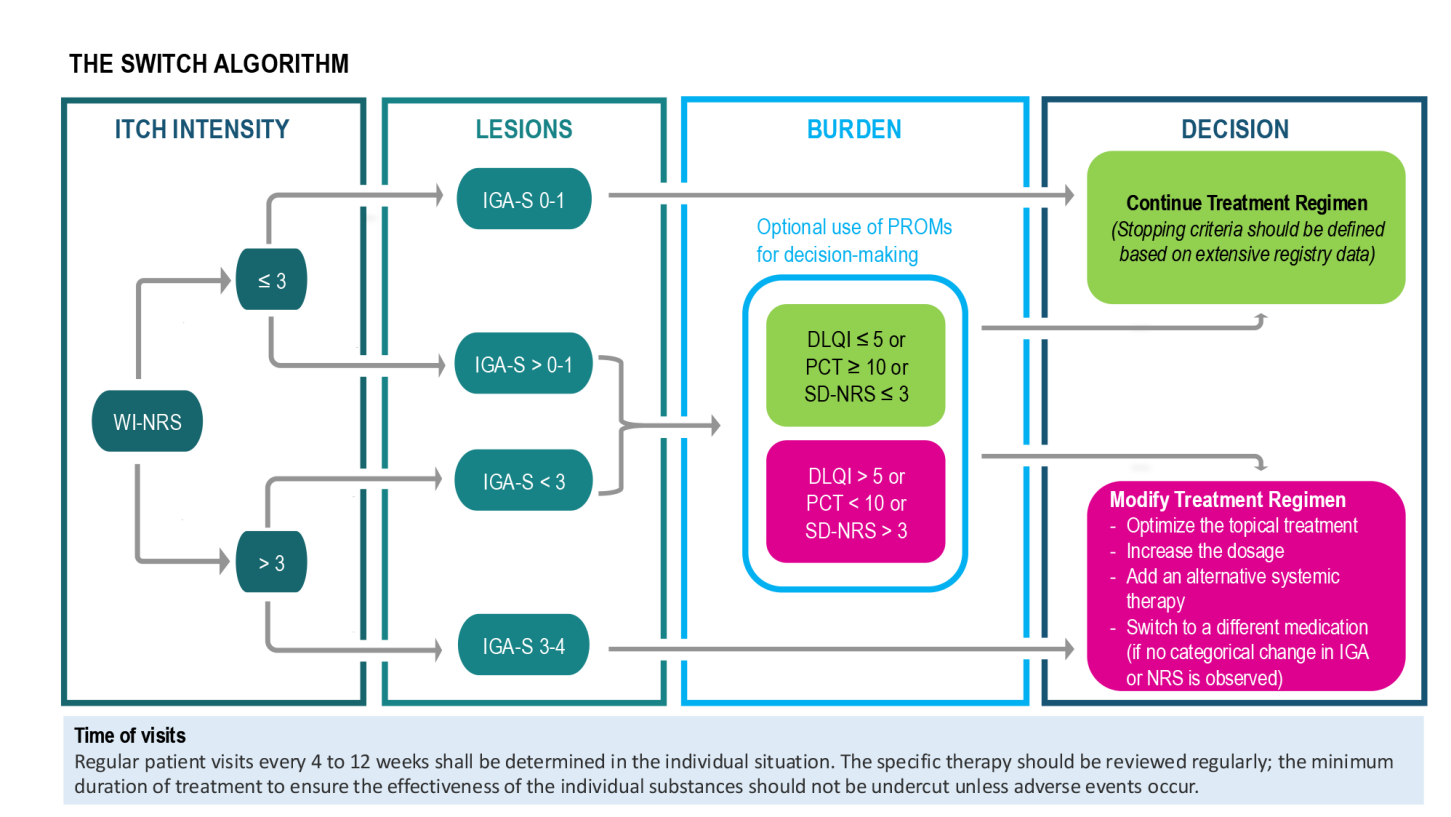
The SWITCH treatment algorithm. At the visit, the worst itch within the past 24 h (WI‐NRS) will first be assessed on a numeric rating scale. Then, the disease severity using the CPG investigator global assessment for the stage (IGA‐CPG stage) is determined by the physician. In intermediate patients (low itch, high disease activity or high itch, low disease activity) additional patient reported outcome measures (PROMs) can be optionally used to determine if patients have an impaired quality of life (using the Dermatology Life Quality Index; DLQI), a controlled disease (using the Prurigo Control Test; PCT) or an impaired sleep (using the sleep disturbance NRS; SD NRS).

In the last three digital meetings (between June 2024 and January 2025), and one anonymous final voting, the EC consented to the T2T criteria (Table 1) and approved the treatment algorithm, which incorporates decision points and corresponding paths (Figure 3).

**TABLE 1:** Identified and consented treat‐to‐target criteria.

T2T criteria	Parameter, recall period (range)	Therapy aim
Itch intensity	WI‐NRS, 24 h (0–10)	Initial: ≤3 Ultimate: 0/1
Pruriginous Lesions	IGA‐CPG stage (0–4)	0/1
Quality of life	DLQI, 1 week (0–30)	Total ≤ 5
Prurigo control test	PCT, 2 weeks (0–20)	Total > 10
Sleep	SD‐NRS, last night (0–10)	Total ≤ 3

Abbreviations: CPG, chronic prurigo; DLQI, Dermatology Life Quality Index; IGA, Investigator Global Assessment; NRS, Numerical Rating Scale; PCT, Prurigo Control Test; SD‐NRS, sleep disturbance NRS; WI, worst itch.

Based on the evaluated RCT data and the results from patient interviews, it was concluded that itch reduction and thereby scratching interruption is the most essential goal of CPG therapy. Although complete itch control (NRS 0/1) is the definitive aim, the initial objective is significant itch decrease (NRS ≤ 3) corresponding to the NRS of patients who considered themselves successfully treated. In addition, open interviews and ranking confirmed a two‐step goal regarding itch relief and itch disappearance. Therefore, a two‐step T2T criterion was selected regarding itch intensity. Clearance of pruriginous lesions was selected as the second most crucial treatment aim. In intermediate patients (low itch, high disease activity or high itch, low disease activity; Figure 3), improvement of quality of life, prurigo control or sleep should also be considered in therapy decisions.

The cut‐off values were based on the interview results (Group B mean/median values of NRS and DLQI), categorization of disease severity (IGA‐CPG‐S) and published data (PCT). For SD‐NRS, no cut‐off value was available for CPG. Based on the distribution in the independent patient cohort reported above, we defined SD‐NRS ≤3 as the threshold for mild sleep disturbances.

Regarding the frequency of re‐evaluation of the T2T criteria, the EC concluded that individual therapy should be regularly re‐assessed based on personal parameters (age, comorbidity, therapy, comedication, patient preferences, adverse events). Still, the efficacy of the specific CPG therapy should be reviewed at least after 3–6 months. This was added to the algorithm, which all members of the EC agreed with by voting.

## DISCUSSION

The treatment landscape for CPG is changing with more therapy options available for this burdensome disease. Accordingly, this group of experts aimed to define criteria for medical care and therapy decisions in CPG. Based on the results from the RCT review, the patient interviews, and a consensus‐building process, the international EC agreed on T2T criteria and a treatment algorithm for CPG, including PN. The T2T criteria are based on the most distressing symptoms of the disease (itch intensity, disease severity, impairment of the quality of life, and sleep disturbance) and the most used instruments for their assessment. The SWITCH treatment algorithm integrates these parameters and their corresponding cut‐off values in a structured decision tree. As the most crucial symptom, itch intensity measured with the WI‐NRS defines the algorithm's first decision point. Based on patients' interviews, WI‐NRS 3 is considered the initial goal, in addition to NRS 0/1 being the ultimate long‐term goal of any therapy. The second important goal is the healing of pruriginous lesions, with the ultimate goal of achieving an IGA‐CPG‐S score of 0/1. Moderate–to‐severe disease severity (IGA‐CPG‐S 3 and 4) indicates treatment or modification of it. Due to limited data availability, we integrated the IGA‐CPG‐S as the primary instrument for lesion assessment. However, alternatives such as PAS may also be valid. Once the two parameters and their potential combinations have been assessed, the algorithm determines whether adjustments to the treatment regimen are necessary. Modifications should be considered if no significant categorical improvement is observed in the IGA‐CPG‐S or the NRS. The individual burden measured by PROMs (PCT, DLQI, and/or SD‐NRS) can refine decision‐making and ensure that the treatment plan aligns with the patient's subjective therapy evaluation and experience. Out of the potential sleep assessment instruments, the SD‐NRS was preferred over the sleep‐NRS because it correlates well with other itch assessment tools and demonstrates good construct validity.^13^ If treatment modification is required, several strategies can be employed, such as improving the topical treatment (e.g. increasing the frequency of application), increasing the dosage of the current systemic therapy, adding an alternative systemic therapy or switching to a different medication.

T2T is a concept that considers both a treatment goal and a time frame for achieving it.^14^ In the case of CPG, the existing traditional therapies were of limited efficacy, and a time frame could not yet be established.^15^ We are in a transition phase of the treatment landscape with the recently approved biologics. Thus, long‐term data are largely missing. Data collected in registries are currently generated and will enable the definition of meaningful time targets, especially for treatment discontinuation. Thus, the SWITCH algorithm contains no recommendations regarding stopping or tapering therapy or estimating the frequency of relapses. Such additions could be considered after obtaining results from pivotal registry studies. In any case, the minimum recommended duration of each treatment must be respected to ensure its effectiveness, unless adverse effects trigger earlier discontinuation. For example, gabapentin should be continued for at least 8 weeks, while biologic therapies require a minimum treatment duration of 3 months before assessing their efficacy adequately.

Effective T2T strategies require therapy goals that are easily measurable and relevant for both patients and clinicians^16^ to achieve improved outcomes.^17^, ^18^, ^19^ Therefore, the results from the patient interviews were crucial in gaining a deeper understanding of patient needs and defining the T2T criteria for CPG. These criteria balance objective clinical markers with patient‐centred outcomes to ensure a holistic approach to treatment decisions. In both RCTs and clinical practice, the intensity of pruritus, measured using the NRS, and the pruriginous lesions, evaluated via the IGA for CPG or with the PAS, are the most frequently utilized instruments. For example, a European study in patients with CPG identified pruritus as the highest burden, followed by the visibility and bleeding of skin lesions.^12^ This relevance was confirmed by both the EC and the prioritization of the patient's burden and treatment aims. As CPG is a highly pruritic disease,^1^ it was not surprising that nearly all patients experience severe itching, followed by scratching and pruriginous lesions as the most severe symptoms. The differences between Group A and Group B indicate that the severity of symptoms is perceived differently depending on patients' disease status. When CPG is active, sleep disturbances are more troubling, and the impact on quality of life is greater than when the disease is successfully treated. Conversely, improved patients fear the progression of CPG and are more concerned about skin lesions and bleeding. This prioritization is reflected in the SWITCH algorithm, which considers, in a first step, itch intensity followed by healing lesions.

In summary, this project successfully defined the first T2T criteria and developed a therapy algorithm, which is subject to further refinement.

### Strengths and limitations

The SWITCH project employed a multi‐phase approach that included qualitative research, regular expert meetings to discuss general therapeutic options, patient interviews and an expert consensus to develop a structured, evidence‐based algorithm for therapy decision‐making. This systematic methodology, combined with the involvement of self‐assessed Group B patients, ensured the creation of a scientifically valid algorithm that addresses the real‐world needs of patients. However, further validation in larger cohorts (with ROC analyses) is needed, including the patient perspective as a reference. This plan will also address the small size of the SD‐NRS subgroups in the current study.

A strength of the study involving patients was that the interviews were conducted using a standardized protocol, ensuring consistency. Open‐ended questions were employed to elicit qualitative and even unexpected answers from patients.^20^ At the same time, closed questions were utilized to obtain patients' ratings on the symptoms observed by the clinicians. The use of cut‐offs for PROMs has limitations and will be substantiated with real‐life patient data.

An additional limitation is that the patient interviews have been performed in one centre with a representative number of Caucasian, skin type II patients. Patients of other ethnicities and skin types with CPG are currently being recruited in multiple centres to confirm patient preferences worldwide.

### Implications for clinical practice

By providing clear T2T criteria for continuing, discontinuing or changing therapy, along with the newly approved PN medicines,^15^, ^21^, ^22^, ^23^, ^24^ the SWITCH algorithm is expected to enhance the outcomes of CPG therapy, improve patients' quality of life and reduce costs, similar to other disease management approaches, such as those in rheumatology.^17^ By adhering to this structured decision‐making process, physicians can ensure that modifications to treatment regimens are evidence‐based and tailored to the patients' needs.

## CONCLUSIONS

Adopting a structured approach with initial assessment of the patient's needs, the SWITCH project defined and achieved expert consensus on T2T criteria for adult patients with mild, moderate and severe CPG, including PN. Based on these validated and easily measurable criteria in the clinical setting, an algorithm was established to enhance CPG therapy outcomes and reduce patient burden.

The SWITCH algorithm offers a valid and systematic approach to evaluating and adjusting treatment based on data reported by patients and validated by experts.

## AUTHOR CONTRIBUTIONS

All authors contributed substantially to the study's conception and/or design, data analysis and interpretation. They also drafted and critically revised the article for important intellectual content and gave final approval of the paper.

## FUNDING INFORMATION

This work was supported by an unrestricted grant from Galderma and by the German Research Foundation (DFG – Deutsche Forschungsgemeinschaft; grant FOR 2690 to SST, grant FOR 5211 to SST).

## CONFLICT OF INTEREST STATEMENT

Lea S. Stahl has no conflict of interest to declare. Claudia Zeidler is an investigator for Novartis, Janssen, Pfizer, UCB, Lilly, AbbVie, Amgen, Celldex, Boehringer Ingelheim, Sanofi, Regeneron, Leo, Galderma and has received speaker honoraria/travel fees from Dermasence, Beiersdorf, Leo, Sanofi, Novartis, Unna Akademie, Allmiral and AbbVie. Joyce Loewenthal has no conflict of interest to declare. Svenja Royeck was an advisor, speaker or investigator for Incyte Inc., Eli Lilly, LEO Pharma and Sanofi/Regeneron outside the submitted work. Matthias Augustin has served as consultant and/or paid speaker for and/or has received research grants and/or honoraries for consulting and/or scientific lectures for and/or got travel expenses reimbursed and/or participated in clinical trials sponsored by companies that manufacture drugs used for the treatment of prurigo including AbbVie, Almirall, Beiersdorf, Eli Lilly, Galderma, Incyte, LEO, Menlo, MSD, Novartis, Pfizer, Regeneron, Sanofi‐Genzyme and Trevi. Robert Bissonnette received grants or contracts or consulting fees from: AbbVie, Aclaris, Alumis, Amgen, Apogee, Arcutis, Areteia, Artax, Attovia, BioMimetix, BlueFin, CARA Therapeutic, Clexio, Dermavant, Eli Lilly, Escient, Fresh Tracks (Brickell), Incyte, Inmagine Bio, Janssen, LEO Pharma, Merck, Opsidio, RAPT Therapeutic, Pfizer, Q32, Sanofi Aventis, Sanofi‐Genzyme, Sitryx, Target RWE, Triveni, Xencor, Zurabio and has stock options of Innovaderm. Sarina Elmariah has served as a consultant, advisory board member or given lectures for Bambusa Therapeutics, Celldex, Disc Medicine, Eli Lilly, Novartis, Pfizer, Regeneron, Sanofi. Brian S. Kim is co‐founder of Alys Pharmaceuticals; he has served as a consultant for 23andMe, ABRAX Japan, AbbVie, Amgen, Attovia Therapeutics, Cara Therapeutics, Clexio Biosciences, Eli Lilly and Company, Escient Pharmaceuticals, Evommune, Galderma, LEO Pharma, Micreos, Novartis, Pfizer, Recens Medical, Regeneron, Sanofi, Septerna, Teva, Trevi Therapeutics, Triveni Bio; he has stock in ABRAX Japan, Alys Pharmaceuticals, Attovia Therapeutics, Locus Biosciences, Recens Medical and Triveni Bio; he holds a patent for the use of JAK1 inhibitors for chronic pruritus. Laurent Misery was a speaker and/or consultant and/or investigator and/or has received research funding from Almirall, Amgen, Clexio, Galderma, GSK, Incyte, Leo Pharma, Lilly, Novartis, Sanofi, Vifor. Hiroyuki Murota was a speaker and/or consultant and/or investigator for Maruho, Sanofi, Regeneron, Kyowa‐Kirin, AbbVie, Lily, Leo, Pfizer, Torii, Kao, Shiseido and Daiichi‐Sankyo Health Care. Hong Liang Tey has no conflict of interest to declare. Elke Weisshaar was consultant/investigator for Galderma, Leo Pharma, Kiniksa, Menlo, Sanofi, Trevi. Gil Yosipovitch was a consultant and investigator for AbbVie, Arcutis, Almiral, Amgen, Attovia Celldex, Escient Health, Eli Lilly, Galderma, LEO Pharma, Merck, Novartis, Pfizer, Regeneron Pharmaceuticals, Inc., Sanofi, Vifor, GSK, Celldex. Franz J. Legat was a speaker and/or consultant and/or investigator and/or has received support to attend scientific meetings from AbbVie, Almirall, Amgen, Celgene, DS Biopharma, Eli Lilly, Galderma, Incyte, Janssen‐Cilag, Kiniksa, Leo Pharma, Menlo Therapeutics, Novartis, Pelpharma, Pfizer, Regeneron, Sanofi, Trevi Therapeutics, Vifor Pharma. Adam Reich is or recently was a speaker and/or advisor for and/or has received research funding from AbbVie, Amgen, AnaptysBio, Arcutis, Argenx, Bausch Health, Biocon, Bioderma, Biothera, Boehringer Ingelheim, Bristol Myers Squibb, Celldex Ther., Celgene, Celltrion, Curateq Biologics, Chema Elektromet, Dice Ther., Galderma, Horizon, Incyte, InflaRx, Janssen, Kiniksa, Leo Pharma, Lilly, Medac, Nektar, Novartis, Numab, Pfizer, Pierre Fabre, Sanofi/Regeneron, Trevi Therapeutics, Sandoz and UCB. Martin Metz has received honoraria as a speaker and/or advisor from AbbVie, Advanz, ALK‐Abello, Allegria, Almirall, Amgen, Argenx, AstraZeneca, Astria, Attovia, Bambusa, Berlin‐Chemie, Blueprint, Celldex, Celltrion, DeepApple, Escient, FatiAbGen, Galderma, Granular, GSK, Incyte, Jasper, Japan Tob. Inc., Lilly, Lycia, Novartis, Pfizer, Pharvaris, Regeneron, Sanofi, Santa Ana Bio, Septerna, Teva, Third Harmonic Bio, Vifor. Sonja Ständer was a speaker and/or consultant and/or investigator and/or has received research funding from Almirall S. A., Amgen Inc., Amgen Europe GmbH, Celldex Therapeutics Inc., Clexio Biosiences Ltd., Focus‐Insight Healthtech Group Co. Ltd., Galderma Laboratorium GmbH, Galderma R&D LLC., Galderma S.A., GSK R&D Ltd., Incyte Corporation, Klirna Biotech Inc., Leo Pharma, Lilly Deutschland GmbH, Novartis Pharma GmbH, Sanofi‐Aventis Deutschland GmbH, Sanofi‐Aventis R&D, Sanofi Genzyme Corporation, TouchIME, Vifor Pharma Deutschland GmbH P. G. Unna Academy.

## ETHICAL APPROVAL

Reviewed and approved by the IRB of Westphalian‐Lippe, Münster, Germany (approval no. 2023‐389‐f‐S).

## ETHICS STATEMENT

This study included only patients who provided written informed consent.

## Supporting information


Table S1.

Table S2.

Table S3.

## Data Availability

The data that support the findings of this study are available from the corresponding author upon reasonable request.
