# Supplementary material for: The SWITCH algorithm: An expert consensus on treat‐to‐target criteria for chronic prurigo
Source: J Eur Acad Dermatol Venereol. 2025 Nov 8;40(7):1185–94. doi: 10.1111/jdv.70171 (PMC13308662; doi:10.1111/jdv.70171)
Supplement: Supplementary file 1 — Table S1. [file JDV-40-1185-s001.docx]

**Supplement Table 1. Review results of randomized controlled trials**

| **ID ClinicalTrials.gov** | **Measurement for Primary Outcome(s)***** | **Measurement for Secondary Outcome(s)***** |
| --- | --- | --- |
| NCT02174419 | NRS | NRS |
|  |  | VRS |
| NCT04183335 | WI-NRS | DLQI |
|  |  | HADS |
|  |  | IGA Prurigo |
|  |  | Skin Pain-NRS |
|  |  | WI-NRS |
| NCT04501679 | WI-NRS | IGA Prurigo |
|  | IGA | PAS |
|  |  | SD NRS |
|  |  | WI-NRS |
| NCT04204616 | *** | AI-NRS |
|  |  | DLQI |
|  |  | EQ-5D |
|  |  | IGA Prurigo |
|  |  | Pain Frequency on a VRS |
|  |  | PGAD |
|  |  | PGAT |
|  |  | SD NRS |
|  |  | Skin Pain-NRS |
|  |  | WI-NRS |
| NCT04501666 | WI-NRS | IGA Prurigo |
|  | IGA | SD NRS |
|  |  | WI-NRS |
| NCT04501679 | WI-NRS | IGA Prurigo |
|  | IGA | PAS |
|  |  | SD NRS |
|  |  | WI-NRS |
| NCT04944862 | *** | WI-NRS |
| NCT05052983 | WI-NRS | IGA Prurigo |
|  | IGA | WI-NRS |
| NCT05061693 | WI-NRS |  |
| NCT05528913 | WI-NRS | DLQI |
|  |  | IGA Prurigo |
|  |  | PAS |
|  |  | PCT |
|  |  | SD NRS |
|  |  | WI-NRS |
| NCT05755438 | WI-NRS | DLQI |
|  |  | EQ-5D-5L |
|  |  | IGA Prurigo |
|  |  | PAS |
|  |  | Skin Pain-NRS |
|  |  | WI-NRS |
|  |  | Skin Pain-NRS |
|  |  | WI-NRS |
| NCT04202679 | WI-NRS | DLQI |
|  |  | HADS |
|  |  | IGA Prurigo |
|  |  | Skin Pain-NRS |
|  |  | Sleep-NRS |
|  |  | WI-NRS |
