# Supplementary material for: The SWITCH algorithm: An expert consensus on treat‐to‐target criteria for chronic prurigo
Source: J Eur Acad Dermatol Venereol. 2025 Nov 8;40(7):1185–94. doi: 10.1111/jdv.70171 (PMC13308662; doi:10.1111/jdv.70171)
Supplement: Supplementary file 2 — Table S2. [file JDV-40-1185-s002.docx]

**Supplement Table 2. Results of the survey between October and November 2023 on initial T2T criteria**

| **In your opinion, which tool(s) for the documentation of itch intensity offers the possibility to make treatment decisions?** | | |
| --- | --- | --- |
|  | **N** | **%** |
| Worst-itch in the last 24h on the numerical rating scale (WI-NRS/24h)^1^ | 12 | 85.7 |
| Average-itch in the last 24h on the numerical rating scale (AI-NRS/24h^1^ | 6 | 42.9 |
| Worst-itch in the last 24h on the visual analogue scale (WI-VAS/24h) ^1^ | 9 | 64.2 |
| Average-itch in the last 24h on the visual analogue scale (AI-VAS/24h) ^1^ | 3 | 21.4 |
| Verbal rating scale (VRS) ^1^ | 7 | 50.0 |
| 5-D Itch Scale^2^ | 3 | 21.4 |
| Itch Severity Scale (ISS)^3^ | 3 | 21.4 |
| Other (specified): | 1 | 7.1 |
| ItchyQoL^4^ | 1 | 7.1 |

1. Storck M, Sandmann S, Bruland P, Pereira MP, Steinke S, Riepe C, Soto-Rey I, Garcovich S, Augustin M, Blome C, Bobko S, Legat FJ, Potekaev N, Lvov A, Misery L, Weger W, Reich A, Şavk E, Streit M, Serra-Baldrich E, Szepietowski JC, Dugas M, Ständer S, Zeidler C. Pruritus Intensity Scales across Europe: a prospective validation study. J Eur Acad Dermatol Venereol. 2021 May;35(5):1176-1185. doi: 10.1111/jdv.17111. Epub 2021 Feb 3. PMID: 33411947.

2. Elman S, Hynan LS, Gabriel V, Mayo MJ. The 5-D itch scale: a new measure of pruritus. Br J Dermatol. 2010 Mar;162(3):587-93. doi: 10.1111/j.1365-2133.2009.09586.x. Epub 2009 Dec 1. PMID: 19995367; PMCID: PMC2875190.

3. Haydek CG, Love E, Mollanazar NK, Valdes Rodriguez R, Lee H, Yosipovitch G, Tharp MD, Hanifin JM, Chen KH, Chen SC. Validation and Banding of the ItchyQuant: A Self-Report Itch Severity Scale. J Invest Dermatol. 2017 Jan;137(1):57-61. doi: 10.1016/j.jid.2016.06.633. Epub 2016 Aug 10. PMID: 27521593.

4. Desai NS, Poindexter GB, Monthrope YM, Bendeck SE, Swerlick RA, Chen SC. A pilot quality-of-life instrument for pruritus. J Am Acad Dermatol. 2008 Aug;59(2):234-44. doi: 10.1016/j.jaad.2008.04.006. Epub 2008 Jun 11. PMID: 18550210.

| **In your opinion, which tool (s) for the documentation of pruriginous lesions offers the possibility to make treatment decisions?** | | |
| --- | --- | --- |
|  | **N** | **%** |
| Counting the pruriginous lesions without any instrument | 2 | 14.3 |
| Estimation of severity without any instrument | 6 | 42.9 |
| Investigator Global Asssement for chronic prurigo (IGA-CPG) activity^1^ | 10 | 71.4 |
| IGA-CPG stage^1^ | 12 | 85.7 |
| Measuring the diameter of the 3 largest nodules on each leg^2^ | 0 | 0 |
| Multidimensional semiquantitative assessment^3^ | 0 | 0 |
| Prurigo Activity and Severity Score (PAS)^4,5^ | 9 | 64.3 |
| Scratch Sign Score (SSS) | 1 | 7.1 |
| Other (specified): | 3 | 14.2 |
| WI-NRS plus nodule estimation | 1 | 7.1 |
| Itch Severity Scale, especially AI-NRS/24h | 1 | 7.1 |
| Measurement of itch intensity | 1 | 7.1 |

1 Zeidler C, Pereira MP, Augustin M, Spellman M, Ständer S. Investigator's Global Assessment of Chronic Prurigo: A New Instrument for Use in Clinical Trials. Acta Derm Venereol. 2021 Feb 17;101(2):adv00401. doi: 10.2340/00015555-3701. PMID: 33236125; PMCID: PMC9366679.

2. Wong SS et al. Arch Dermatol 2000; 136: 807-8.

3. Mazza M et al. J Clin Pharm Ther 2013;38:16-8

4. Pölking J, Zeidler C, Schedel F, Osada N, Augustin M, Metze D, Pereira MP, Yosipovitch G, Bernhard JD, Ständer S. Prurigo Activity Score (PAS): validity and reliability of a new instrument to monitor chronic prurigo. J Eur Acad Dermatol Venereol. 2018 Oct;32(10):1754-1760. doi: 10.1111/jdv.15040. Epub 2018 Jun 7. PMID: 29729201.

5. Zeidler C, Stander S, Rhoten S, Wratten S, Zhang D, Msihid J, Brookes E, Thomas R, Bahloul D. Validation of a scoring algorithm for the clinician-reported outcome tool 'prurigo activity and severity (PAS)' based on clinical studies of dupilumab in adults with prurigo Nodularis. J Eur Acad Dermatol Venereol. 2024 Oct;38(10):1954-1964. doi: 10.1111/jdv.19961. Epub 2024 Apr 2. PMID: 38563058.
