# Supplementary material for: The SWITCH algorithm: An expert consensus on treat‐to‐target criteria for chronic prurigo
Source: J Eur Acad Dermatol Venereol. 2025 Nov 8;40(7):1185–94. doi: 10.1111/jdv.70171 (PMC13308662; doi:10.1111/jdv.70171)
Supplement: Supplementary file 3 — Table S3. [file JDV-40-1185-s003.docx]

**Supplement Table 3. Demographic and clinical characteristics of the CPG patients.** Group A (Therapy Need): patients with moderate-to-severe CPG (IGA 3/4) and severe intense itch (WI-NRS ≥ 7). Group B (Therapy Success): patients with CPG considering themselves successfully treated without predetermination of IGA or NRS.

Abbreviations: CPG: chronic prurigo; WI- NRS: worst itch intensity numerical rating scale; IGA-CPG-A/S: Investigator Global Assessment for CPG–activity/stage; PCT: Prurigo Control Test; DLQI: Dermatology Life Quality Index; SD: Standard deviation; Q1/3, first/third quartile; TUE: Technical University Entry.

Other therapies: Opioid receptor modulators (Naloxone/Naltrexone);

|  | **All**  **n = 171**  **n (%)** | **Group A**  **n=96**  **n (%)** | **Group B**  **n=75**  **n (%)** |
| --- | --- | --- | --- |
| *Demographics*  Sex, *n (%)*  Female  Male  Age (years)  Mean ± SD  Median [Q1;Q3]  Ethnicity, *n (%)*  Caucasian  Asian  African American  Others  *Disease characteristics*  Skin type, *n (%)*  I  II  III  IV  V  VI  Age at onset, years  Mean ± SD  Median [Q1;Q3]  WI-NRS 24 h  Mean ± SD  Median [Q1;Q3]  IGA­CPG­S  Mean ± SD  Median [Q1;Q3]  IGA-CPG-A  Mean ± SD  Median [Q1;Q3]  DLQI  Mean ± SD  Median [Q1;Q3]  PCT  Mean ± SD  Median [Q1;Q3] | 85 (49.7)  86 (50.3)  64.6 ± 13.3  65 (57.73)  169 (98.8)  2 (1.2)  0  0  9 (5.3)  149 (87.1)  11 (6.4)  2 (1.2)  0  0  54.9 ± 6.1  56 (45;66)  5.8 ± 3.1  7 (3;8)  2.3 ± 1.1  3 (1;3)  2.3 ± 1.1  3 (1;3)  8.3 ± 6  8 (4;12)  9.5 ± 5.8  9 (4;15) | 48 (50)  48 (50)  65 ± 14.1  65 (56;76.8)  95 (99)  1 (1)  0  0  4 (4.2)  83 (86.5)  8 (8.3)  1 (1)  0  0  56.5 ± 15.8  57.5 (44.3;67)  8.2 ± 1.1  8 (7;9)  3.1 ± 0.3  3 (3;3)  3.1 ± 0.3  3 (3;3)  11.7 ± 5.5  10.5 (8;15)  5.1 ± 3.2  5 (2.3;7.8) | 37 (49.3)  38 (50.7)  64 ± 12.3  64.8 (58.1;71.3)  75 (98.7)  1 (1.3)  0  0  5 (6.7)  66 (88)  3 (4)  1 (1.3)  0  0  52.9 ± 16.4  53 (45;63)  2.7 ± 1.6  3 (2;4)  1.3 ± 0.9  1 (1;2)  1.3 ± 0.9  1 (1;2)  3.9 ± 3.1  3 (1;6)  15.1 ± 2.6  15 (13;17) |
